# Supplementary figures and images for: Validation of deep learning enabled software MetronMind to measure vertebral heart size and vertebral left atrial size in dogs
Source: PLoS One. 2026 Jun 30;21(6):e0339253. doi: 10.1371/journal.pone.0339253 (PMC13318042; doi:10.1371/journal.pone.0339253)

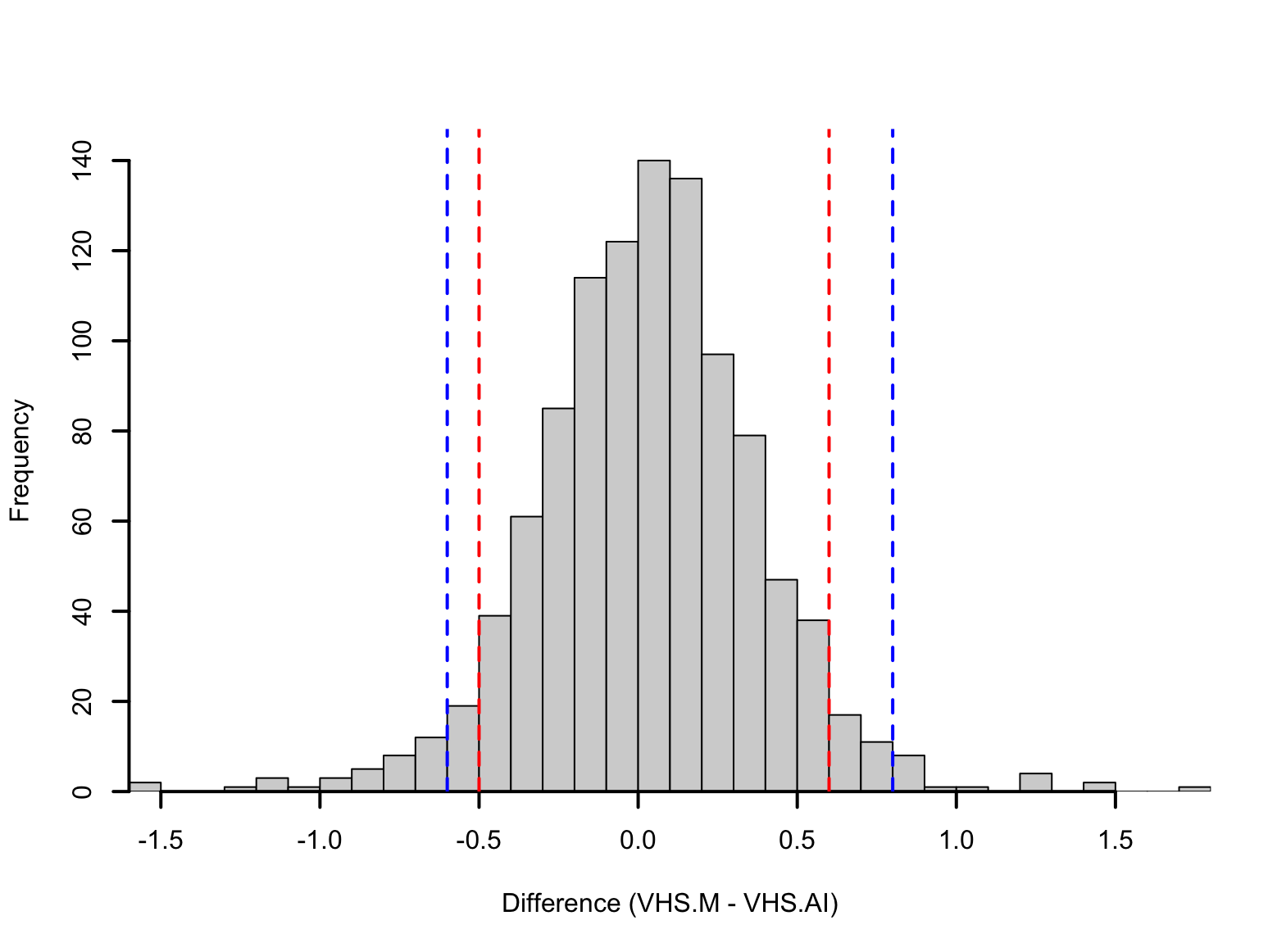

Supplement: S1 Fig — The red lines indicate the 5th percentile and 95th percentile. The blue dashed lines represent the 2.5th percentile and the 97.5th percentile. (TIF) [file pone.0339253.s003.tif]

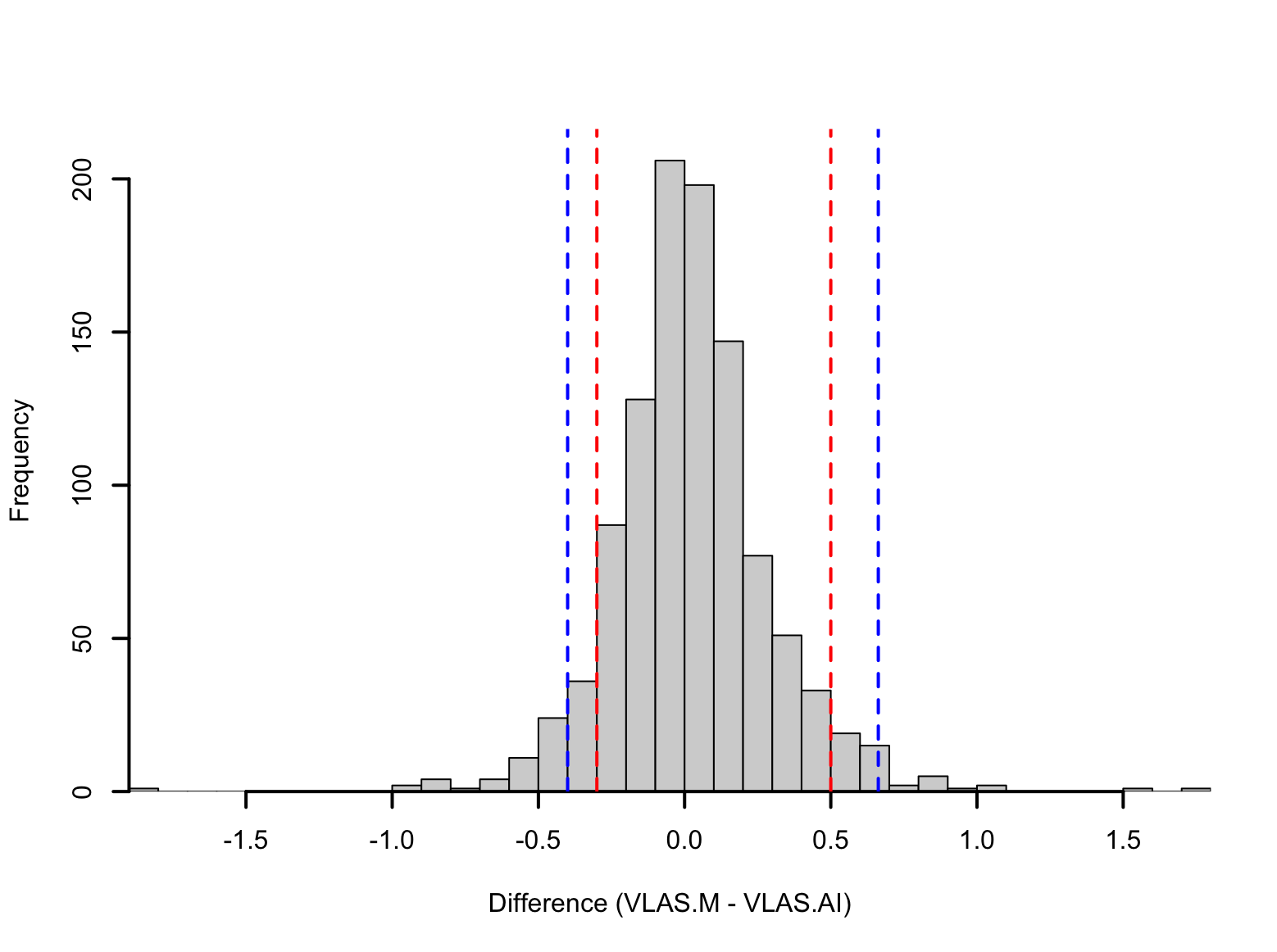

Supplement: S2 Fig — The red lines indicate the 5th percentile and 95th percentile. The blue dashed lines represent the 2.5th percentile and the 97.5th percentile. (TIF) [file pone.0339253.s004.tif]
