# Supplementary material for: Validation of deep learning enabled software MetronMind to measure vertebral heart size and vertebral left atrial size in dogs
Source: PLoS One. 2026 Jun 30;21(6):e0339253. doi: 10.1371/journal.pone.0339253 (PMC13318042; doi:10.1371/journal.pone.0339253)
Supplement: S1 File — (PDF) [file pone.0339253.s005.pdf]

```
##########################################################################
##############
############# NOTES
################################################################
##########################################################################
##############
# Purpose: Script for stats on MetronMind data
# Author: Alice Watson
# Date: 13th October 2025


library(mcr) ##For passing bablok
library(blandr) ##For bland altman analysis
library(dplyr)
library(tidyr)
library(ggplot2)
library(broom)
library(purrr)
library(tidyr)


############# Save and Load Workspace
##############################################
##########################################################################
##############
#save workspaces, still need to run libraries and also setwd when
loading
rm(list=ls())


mainDir <-"/Users/alicewatson/Dropbox/Alice&Sonya_2024/#2MetronmindAI/
AliceWorkingFiles"
setwd(mainDir)
excellentDir <-"/Users/alicewatson/Dropbox/Alice&Sonya_2024/
#2MetronmindAI/AliceWorkingFiles/ExcellentFigs"
notPoorDir <-"/Users/alicewatson/Dropbox/Alice&Sonya_2024/
#2MetronmindAI/AliceWorkingFiles/ExcludePoorFigs"

#Loading workspace - remember to load correct working directory first!
setwd(mainDir)
load("Metron.RData")
save.image("Metron.RData")

#####FUNCTIONS#####

report_cor <- function(data, x, y) {
  data %>%
    summarise(cor_test = list(cor.test({{x}}, {{y}}))) %>%
    mutate(tidy_res = map(cor_test, tidy)) %>%
```

```
    unnest(tidy_res) %>%
    mutate(
      report = sprintf(
        "%.3f (%.3f to %.3f), p = %.3f",
        estimate, conf.low, conf.high, p.value
      )
    ) %>%
    pull(report)
}

report_pb_para <- function(pb_para) {
  # Extract Intercept
  intercept <- pb_para["Intercept", "EST"]
  intercept_LCI <- pb_para["Intercept", "LCI"]
  intercept_UCI <- pb_para["Intercept", "UCI"]

  intercept_report <- sprintf(
    "Intercept = %.2f (%.2f to %.2f)",
    intercept, intercept_LCI, intercept_UCI
  )

  # Extract Slope
  slope <- pb_para["Slope", "EST"]
  slope_LCI <- pb_para["Slope", "LCI"]
  slope_UCI <- pb_para["Slope", "UCI"]

  slope_report <- sprintf(
    "Slope = %.2f (%.2f–%.2f)",
    slope, slope_LCI, slope_UCI
  )

  tibble(
    slope_report = slope_report,
    intercept_report = intercept_report
  )
}

report_bland_altman <- function(x, y) {
  # Run Bland–Altman statistics (fixed 95% CI)
  # Remove missing values
  valid <- complete.cases(x, y)
  x <- x[valid]
  y <- y[valid]
  n_pairs <- length(x)
  #Run Bland-Altman
  ba <- blandr.statistics(x, y, sig.level = 0.95)

  # Extract relevant stats
  bias <- ba$bias
  bias_low <- ba$biasLowerCI
```

```
  bias_high <- ba$biasUpperCI

  loa_upper <- ba$upperLOA
  loa_upper_low <- ba$upperLOA_lowerCI
  loa_upper_high <- ba$upperLOA_upperCI

  loa_lower <- ba$lowerLOA
  loa_lower_low <- ba$lowerLOA_lowerCI
  loa_lower_high <- ba$lowerLOA_upperCI

  # Format output text
  bias_report <- sprintf(
    "Bland-Altman bias = %.3f (%.3f to %.3f)",
    bias, bias_low, bias_high
  )

  loa_report <- sprintf(
    "Bland-Altman upper LoA = %.3f (%.3f to %.3f), lower LoA = %.3f
(%.3f to %.3f)",
    loa_upper, loa_upper_low, loa_upper_high,
    loa_lower, loa_lower_low, loa_lower_high
  )

  # Return as tibble
  tibble(
    c(n_pairs = n_pairs,
    bias_report = bias_report,
    loa_report = loa_report)
  )
}


##Read in MetronMind Data
dat_full <- "MetronMind.csv"


#####################################
## read in the data in .csv format
#####################################
full <- read.csv(paste0(mainDir, "/", dat_full), header = T,
na.strings = c("","NA","na","0"))

##Notes about data:
#VHS and VLAS-O are original VLAS by submitting Dr
#Weight is in Kg
#Age in in years
#OverallRLVHSlandmarks: Right lateral: VHS landmarks overall
assessment E=excellent, D=diagnostic, P=poor, NA=cant measure
#OverallRLVLASlandmarks: Right Lateral: VLAS landmarks overall
assessment E=excellent, D=diagnostic, P=poor, NA=cant measure
```

```
#DorsalVC: Dorsal Vena cava insertion for VLAS
#VentralVC: Ventral VC for VHS
#CranialLV: LV crainal aspect for VHS
#CrT4: Cranial aspect T4
#AllVert:Vertebral bodies overall
#VHS-T: VHS-human_traditional
#VLAS-T: VLAS-human traditional
#VHS-M: VHS-Human_modified
#VLAS-M: VLAS-human traditional
#VLAS-Metron
#VHS-Metron: VHS-AI-Metron--3 (Aug 2022 final)

#Convert character to categorical (factor)
full <- full %>%
  mutate(across(where(is.character), as.factor))



attach(full)
####Demographic info####
#Summarise breeds
breed_summary <- full %>%
  group_by(Breed) %>%
  summarise(Count = n()) %>%
  mutate(Percent = 100 * Count / sum(Count)) %>%
  arrange(desc(Count))%>%
  mutate(Percent = round(Percent, 1))  # one decimal place

breed_summary

write.csv(breed_summary, "breed_summary.csv", row.names = FALSE)

##
summary(full$Sex)

shapiro.test(full$Age) #Not normal distribution as p-value = 2.205e-07
summary(full$Age)

hist(full$Weight)
shapiro.test(full$Weight) #Not normal distribution as p-value <
2.2e-16
summary(full$Weight)


summary(full$VHS.T)
summary(full$VHS.M)
summary(full$VHS.Metron)

summary(full$VLAS.T)
summary(full$VLAS.M)
```

```
summary(full$VLAS.Metron)

hist(full$VHS.T)
shapiro.test(full$VHS.T)#Not normal distribution as p-value = 
1.951e-14
hist(full$VLAS.T)
shapiro.test(full$VLAS.T)#Not normal distribution as p-value = 
3.412e-15

summary(full$OverallRLVHSlandmarks)
summary(full$OverallRLVLASlandmarks)


LandmarksVHS <- full %>%
  group_by(OverallRLVHSlandmarks) %>%
  summarise(Count = n()) %>%
  mutate(Percent = 100 * Count / sum(Count)) %>%
  arrange(desc(Count))%>%
  mutate(Percent = round(Percent, 1))  # one decimal place
LandmarksVHS

LandmarksVLAS <- full %>%
  group_by(OverallRLVLASlandmarks) %>%
  summarise(Count = n()) %>%
  mutate(Percent = 100 * Count / sum(Count)) %>%
  arrange(desc(Count))%>%
  mutate(Percent = round(Percent, 1))  # one decimal place
LandmarksVLAS

IndLandmark <- full %>%
  pivot_longer(cols = c(Carina, LVapex, DorsalVC, VentralVC, 
CranialLV, CrT4, AllVert),
               names_to = "Landmark",
               values_to = "Position") %>%
  group_by(Landmark, Position) %>%
  summarise(Count = n(), .groups = "drop_last") %>%
  mutate(Percent = round(100 * Count / sum(Count), 1)) %>%
  mutate(Label = paste0(Count, " (", Percent, "%)")) %>%
  select(Landmark, Position, Label) %>%
  pivot_wider(names_from = Position, values_from = Label) %>%
  arrange(Landmark)
#Print table
write.csv(IndLandmark, "Table1.csv", row.names = FALSE)

#####VHS-traditional vs modified - Fig 1 #####
##Fig 1A Passing Bablok analysis for comparison of methods VHS 
Traditional vs Modified
#Remove any rows with NA
cor.test(full$VHS.T,full$VHS.M)
    #r=0.993 CI 0.993-0.994
```

```
PB.reg<- mcreg(full$VHS.T,full$VHS.M, method.reg = "PaBa",na.rm=T)
PB.reg@para
  #                EST SE        LCI        UCI
  #Intercept -0.58125 NA -0.6857143 -0.4969061
  #Slope      1.06250 NA  1.0540541  1.0714286

plot(PB.reg, add.grid=F,x.lab = "VHS-T", y.lab = "VHS-M")

tiff("Figure1A_VHSmanual.tiff", width = 12, height = 12,units = "cm",
res=300,pointsize = 10)
MCResult.plot(PB.reg, equal.axis = TRUE, x.lab = "VHS-T", y.lab =
"VHS-M",
              ci.area = TRUE,
              main = "", sub = "", add.grid = FALSE, points.cex = 1,
              add.legend = T, add.cor = T)
dev.off()

#BlandAltman Analysis
attach(full)
blandr.statistics(VHS.T,VHS.M, sig.level=0.95)



#Save file with Fig1B
tiff("Figure1B_VHSmanual.tiff", width = 12, height = 12,units = "cm",
res=600,pointsize = 1)
grid::grid.draw(blandr.draw(VHS.T,VHS.M,method1name = "VHS-
T",method2name = "VHS-M",
                            plotTitle = "")+
                  theme_classic() +
                  labs(x = "Mean VHS", y= "Difference VHS.T-VHS.M"))
dev.off()



#####Repeat for VHS-M vs AI - Fig 3####
##Fig 3A Passing Bablok analysis for comparison of methods VHS
Modified vs AI
cor.test(full$VHS.M,full$VHS.Metron)##corr = 0.947, p-value < 2.2e-16,
n= 1055
PB.reg_hai<- mcreg(full$VHS.M,full$VHS.Metron, method.reg =
"PaBa",na.rm=T)
PB.reg_hai@para



tiff("Figure3A_VHSai.tiff", width = 12, height = 12,units = "cm",
res=300,pointsize = 10)
MCResult.plot(PB.reg_hai, equal.axis = TRUE, x.lab = "VHS-M", y.lab =
```

```
"VHS-AI",
              ci.area = TRUE,
              main = "", sub = "", add.grid = FALSE, points.cex = 1,
              add.legend = T, add.cor = T)
dev.off()

##Bland Altman
blandr.statistics(VHS.M,VHS.Metron, sig.level=0.95)



#Save file with Fig3B
tiff("Figure3B_VHSmetron.tiff", width = 12, height = 12,units = "cm",
res=600,pointsize = 1)
grid::grid.draw(blandr.draw(VHS.M,VHS.Metron,method1name = "VHS-
M",method2name = "VHS-AI",
                            plotTitle = "")+
                  theme_classic() +
                  labs(x = "Mean VHS", y= "Difference VHS.M-VHS.AI"))
dev.off()


####Compare VHS-O to VHS-M - Fig 5#####
##Fig 5A Passing Bablok analysis for comparison of methods VHS
Traditional vs Modified
#Remove any rows with NA


cor.test(full$VHS.M,full$VHS.O) ##r=0.947, p< 2.2e-16, n=1054
PB.rego<- mcreg(full$VHS.M,full$VHS.O, method.reg = "PaBa",na.rm=T)
PB.rego@para

plot(PB.rego, add.grid=F,x.lab = "VHS-M", y.lab = "VHS-O")

tiff("Figure5A_VHSoriginal.tiff", width = 12, height = 12,units =
"cm", res=300,pointsize = 10)
MCResult.plot(PB.rego, equal.axis = TRUE, x.lab = "VHS-M", y.lab =
"VHS-O",
              ci.area = TRUE,
              main = "", sub = "", add.grid = FALSE, points.cex = 1,
              add.legend = T, add.cor = T)
dev.off()

#BlandAltman Analysis Fig 5B

blandr.statistics(full$VHS.M,full$VHS.O, sig.level=0.95)
blandr.draw(full$VHS.M,full$VHS.O,method1name = "VHS-M",method2name =
"VHS-O",
               plotTitle = "")+
  theme_classic() +
```

```
  labs(x = "Mean VHS", y= "Difference VHS.M-VHS.O")

tiff("Figure5B_VHSoriginal.tiff", width = 12, height = 12,units =
"cm", res=600,pointsize = 1)
grid::grid.draw(blandr.draw(full$VHS.M,full$VHS.O,method1name = "VHS-
M",method2name = "VHS-O",
                            plotTitle = "")+
                  theme_classic() +
                  labs(x = "Mean VHS", y= "Difference VHS.M-VHS.O"))
dev.off()


###repeat for VLAS T vs M - Fig 2####
##Fig 2A Passing Bablok analysis for comparison of methods VLAS
Traditional vs Modified
#Remove any rows with NA


plot(full$VLAS.T,full$VLAS.M,main="compare scatter")
cor.test(full$VLAS.T,full$VLAS.M) # Corr = 0.9735214 CI 0.9701740
0.9764976 , p<2.2e-16
report_cor(full, VLAS.T,VLAS.M)

pb.reg_l<- mcreg(full$VLAS.T,full$VLAS.M, method.reg = "PaBa",na.rm=T)
pb.reg_l@para
report_pb_para(pb.reg_l@para)


tiff("Figure2A_VLAS-manual.tiff", width = 12, height = 12,units =
"cm", res=300,pointsize = 10)
MCResult.plot(pb.reg_l, equal.axis = TRUE, x.lab = "VLAS-T", y.lab =
"VLAS-M",
              ci.area = TRUE,
              main = "", sub = "", add.grid = FALSE, points.cex = 1,
              add.legend = T, add.cor = T)
dev.off()

#BlandAltman Analysis
attach(full)
blandr.statistics(VLAS.T,VLAS.M, sig.level=0.95)
report_bland_altman(VLAS.T,VLAS.M)

str(ba)

blandr.draw(VLAS.T,VLAS.M,method1name = "VLAS-T",method2name = "VLAS-
M",
               plotTitle = "")+
  theme_classic() +
  labs(x = "Mean VLAS", y= "Difference VLAS.T-VLAS.M")
```

```
#Save file with Fig2B
tiff("Figure2B_VLAS-manual.tiff", width = 12, height = 12,units =
"cm", res=600,pointsize = 1)
grid::grid.draw(blandr.draw(VLAS.T,VLAS.M,method1name = "VLAS-
T",method2name = "VLAS-M",
                            plotTitle = "")+
                  theme_classic() +
                  labs(x = "Mean VLAS", y= "Difference VLAS.T-
VLAS.M"))
dev.off()


####Repeat for VLAS-M vs VLAS-AI - Fig 4####
##Fig 4A Passing Bablok analysis for comparison of methods VHS
Modified vs AI
cor.test(full$VLAS.M,full$VLAS.Metron)##corr = 0.811, p-value <
2.2e-16, n= 1055
report_cor(full,VLAS.M,VLAS.Metron)

PB.reg_lai<- mcreg(full$VLAS.M,full$VLAS.Metron, method.reg =
"PaBa",na.rm=T)
PB.reg_lai@para
report_pb_para(PB.reg_lai@para)



tiff("Figure4A_VLASai.tiff", width = 12, height = 12,units = "cm",
res=300,pointsize = 10)
MCResult.plot(PB.reg_lai, equal.axis = TRUE, x.lab = "VLAS-M", y.lab =
"VLAS-AI",
              ci.area = TRUE,
              main = "", sub = "", add.grid = FALSE, points.cex = 1,
              add.legend = T, add.cor = T)
dev.off()

##Bland Altman
blandr.statistics(VLAS.M,VLAS.Metron, sig.level=0.95)
report_bland_altman(VLAS.M,VLAS.Metron)

#Save file with Fig4B
tiff("Figure4B_VLASmetron.tiff", width = 12, height = 12,units = "cm",
res=600,pointsize = 1)
grid::grid.draw(blandr.draw(VLAS.M,VLAS.Metron,method1name = "VLAS-
M",method2name = "VLAS-AI",
                            plotTitle = "")+
                  theme_classic() +
                  labs(x = "Mean VLAS", y= "Difference VLAS.M-
VLAS.AI"))
dev.off()
```

```
####Compare VLAS-O to VLAS-M - Fig 6#####
##Fig 6A Passing Bablok analysis for comparison of methods VHS
Traditional vs Modified
#Remove any rows with NA


cor.test(full$VLAS.M,full$VLAS.O) ##r=0.844, p< 2.2e-16, n=820
report_cor(full,VLAS.M,VLAS.O)
PB.reglo<- mcreg(full$VLAS.M,full$VLAS.O, method.reg = "PaBa",na.rm=T)
PB.reglo@para
report_pb_para(PB.reglo@para)

tiff("Figure6A_VLASoriginal.tiff", width = 12, height = 12,units =
"cm", res=300,pointsize = 10)
MCResult.plot(PB.reglo, equal.axis = TRUE, x.lab = "VLAS-M", y.lab =
"VLAS-O",
              ci.area = TRUE,
              main = "", sub = "", add.grid = FALSE, points.cex = 1,
              add.legend = T, add.cor = T)
dev.off()

#BlandAltman Analysis Fig 6B
blandr.statistics(full$VLAS.M,full$VLAS.O, sig.level=0.95)
report_bland_altman(VLAS.M,VLAS.O)
blandr.draw(full$VLAS.M,full$VLAS.O,method1name = "VLAS-M",method2name
= "VLAS-O",
            plotTitle = "")+
  theme_classic() +
  labs(x = "Mean VLAS", y= "Difference VLAS.M-VLAS.O")

tiff("Figure6B_VLASoriginal.tiff", width = 12, height = 12,units =
"cm", res=600,pointsize = 1)
grid::grid.draw(blandr.draw(full$VLAS.M,full$VLAS.O,method1name =
"VLAS-M",method2name = "VLAS-O",
                            plotTitle = "")+
                  theme_classic() +
                  labs(x = "Mean VLAS", y= "Difference VLAS.M-
VLAS.O"))
dev.off()

####Left lateral VHS and VLAS - Fig 7 (T vs M)####
##Fig 7A Passing Bablok analysis for comparison of methods Left
lateral VHS Modified vs traditional
#Remove any rows with NA

attach(full)
cor.test(full$LL_VHS.human.traditional,full$LL.VHS.human.modified)
```

```
  ##r=0.942 (0.9305078 0.951615), p< 2.2e-16, n=443
PB.regLL<-
mcreg(full$LL_VHS.human.traditional,full$LL.VHS.human.modified,
method.reg = "PaBa",na.rm=T)
PB.regLL@para
  #             EST SE       LCI        UCI
  #Intercept -0.46 NA -0.593750 -0.2672059
  #Slope      1.05 NA  1.029412  1.0625000
tiff("Figure7A_LLVHShuman.tiff", width = 12, height = 12,units = "cm",
res=300,pointsize = 10)
MCResult.plot(PB.regLL, equal.axis = TRUE, x.lab = "LL-VHS-T", y.lab =
"LL-VHS-M",
              ci.area = TRUE,
              main = "", sub = "", add.grid = FALSE, points.cex = 1,
              add.legend = T, add.cor = T)
dev.off()

#BlandAltman Analysis Fig 7B
blandr.statistics(full$LL_VHS.human.traditional,full$LL.VHS.human.modi
fied, sig.level=0.95)
# t = -4.3768, df = 444, p-value = 1.503e-05
#SE 0.01956195 Standard error for limits of agreement:  0.03345641
#Bias:  -0.08561798  ( -0.1240635  to  -0.04717245 )
#ULoA:   0.7231954  ( 0.6574428  to  0.788948 )
#LLoA:   -0.8944314  ( -0.960184  to  -0.8286788 )

tiff("Figure7B_LLVHSoriginal.tiff", width = 12, height = 12,units =
"cm", res=600,pointsize = 1)
grid::grid.draw(blandr.draw(full$LL_VHS.human.traditional,full$LL.VHS.
human.modified,method1name = "LL-VHS-T",method2name = "LL-VHS-M",
                            plotTitle = "")+
                  theme_classic() +
                  labs(x = "Mean LL.VHS", y= "Difference LL.VHS.T-
LL.VHS.M"))
dev.off()

##Fig 7C Passing Bablok analysis for comparison of methods Left
lateral VLAS Modified vs traditional
#Remove any rows with NA


cor.test(full$LL.VLAS.human.Traditional,full$LL.VLAS.human.modified)
##r=0.959, p< 2.2e-16, n=438
report_cor(full,LL.VLAS.human.Traditional,LL.VLAS.human.modified)

PB.regLLvlas<-
mcreg(full$LL.VLAS.human.Traditional,full$LL.VLAS.human.modified,
method.reg = "PaBa",na.rm=T)
report_pb_para(PB.regLLvlas@para)
```

```
tiff("Figure7C_LLVLAShuman.tiff", width = 12, height = 12,units =
"cm", res=300,pointsize = 10)
MCResult.plot(PB.regLLvlas, equal.axis = TRUE, x.lab = "LL-VLAS-T",
y.lab = "LL-VLAS-M",
              ci.area = TRUE,
              main = "", sub = "", add.grid = FALSE, points.cex = 1,
              add.legend = T, add.cor = T)
dev.off()

#BlandAltman Analysis Fig 7D
blandr.statistics(full$LL.VLAS.human.Traditional,full$LL.VLAS.human.mo
dified, sig.level=0.95)
report_bland_altman(LL.VLAS.human.Traditional,LL.VLAS.human.modified)
  #t = 3.7072, df = 438, p-value = 0.0002365, n=439
  #Bias: 0.02505695  ( 0.01177273  to  0.03834116 )
  #ULoA: 0.3026282  ( 0.2799082  to  0.3253481 )
  #LLoA: -0.2525143  ( -0.2752342  to  -0.2297943 )
tiff("Figure7D_LLVLASoriginal.tiff", width = 12, height = 12,units =
"cm", res=600,pointsize = 1)
grid::grid.draw(blandr.draw(full$LL.VLAS.human.Traditional,full$LL.VLA
S.human.modified,method1name = "LL-VLAS-T",method2name = "LL-VLAS-M",
                            plotTitle = "")+
                  theme_classic() +
                  labs(x = "Mean LL.VLAS", y= "Difference LL.VLAS.T-
LL.VLAS.M"))
dev.off()

### R lateral Vs L Lateral- Fig8 VHS and 9 VLAS####
##Fig 8A Passing Bablok analysis for comparison of methods Left
lateral VHS Modified
#Remove any rows with NA


cor.test(full$VHS.M,full$LL.VHS.human.modified) ##r=0.871, p< 2.2e-16,
n=443
report_cor(full, VHS.M, LL.VHS.human.modified)
PB.regRL<- mcreg(full$VHS.M,full$LL.VHS.human.modified, method.reg =
"PaBa",na.rm=T)

report_pb_para(PB.regRL@para)

tiff("Figure8A_RLvsLLVHShuman.tiff", width = 12, height = 12,units =
"cm", res=300,pointsize = 10)
MCResult.plot(PB.regRL, equal.axis = TRUE, x.lab = "RL-VHS-M", y.lab =
"LL-VHS-M",
              ci.area = TRUE,
              main = "", sub = "", add.grid = FALSE, points.cex = 1,
              add.legend = T, add.cor = T)
dev.off()
```

```
#BlandAltman Analysis Fig 8B
blandr.statistics(full$VHS.M,full$LL.VHS.human.modified,
sig.level=0.95)
report_bland_altman(VHS.M,LL.VHS.human.modified)

tiff("Figure8B_RLvsLLVHShuman.tiff", width = 12, height = 12,units =
"cm", res=600,pointsize = 1)
grid::grid.draw(blandr.draw(full$VHS.M,full$LL.VHS.human.modified,meth
od1name = "VHS-M-Right",method2name = "VHS-M-Left",
                            plotTitle = "")+
                  theme_classic() +
                  labs(x = "Mean VHS", y= "Difference VHS.Right-
VHS.Left"))
dev.off()

##Fig 9A Passing Bablok analysis for comparison of methods Left
lateral VLAS Modified vs traditional
#Remove any rows with NA


cor.test(full$VLAS.M,full$LL.VLAS.human.modified) ##r=0.726, p<
2.2e-16, n=437
report_cor(full, VLAS.M,LL.VLAS.human.modified)
PB.regRLvlas<- mcreg(full$VLAS.M,full$LL.VLAS.human.modified,
method.reg = "PaBa",na.rm=T)
report_pb_para(PB.regRLvlas@para)


tiff("Figure9A_RLvsLLVLAShuman.tiff", width = 12, height = 12,units =
"cm", res=300,pointsize = 10)
MCResult.plot(PB.regRLvlas, equal.axis = TRUE, x.lab = "RL-VLAS-M",
y.lab = "LL-VLAS-M",
              ci.area = TRUE,
              main = "", sub = "", add.grid = FALSE, points.cex = 1,
              add.legend = T, add.cor = T)
dev.off()

#BlandAltman Analysis Fig 9B
blandr.statistics(full$VLAS.M,full$LL.VLAS.human.modified,
sig.level=0.95)
report_bland_altman(VLAS.M,LL.VLAS.human.modified)

blandr.draw(full$VLAS.M,full$LL.VLAS.human.modified,method1name = "RL-
VLAS-M",method2name = "LL-VLAS-M",
            plotTitle = "")+
  theme_classic() +
  labs(x = "Mean VLAS", y= "Difference RL.VLAS-LL.VLAS")

tiff("Figure9B_RLvsLLVLAShuman.tiff", width = 12, height = 12,units =
"cm", res=600,pointsize = 1)
```

```
grid::grid.draw(blandr.draw(full$VLAS.M,full$LL.VLAS.human.modified,me
thod1name = "VLAS-M-Right",method2name = "VLAS-M-Left",
                            plotTitle = "")+
                  theme_classic() +
                  labs(x = "Mean VLAS", y= "Difference VLAS.Right-
VLAS.Left"))
dev.off()




####Now analyse only excellent rads####
setwd(excellentDir)
#VHS n=161
excellentVHSrlE<- full%>%
  filter(OverallRLVHSlandmarks == "E")
#VLAS n=161
excellentVLASrlE<- full%>%
  filter(OverallRLVLASlandmarks == "E")
#####VHS-traditional vs modified - Fig 1 #####
##Fig 1A Passing Bablok analysis for comparison of methods VHS
Traditional vs Modified
#Remove any rows with NA
cor.test(excellentVHSrlE$VHS.T,excellentVHSrlE$VHS.M)
  #r= 0.992 CI 0.989-0.994, t = 97.437, df = 159, p-value < 2.2e-16
report_cor(excellentVHSrlE,VHS.T,VHS.M)
PB.rege<- mcreg(excellentVHSrlE$VHS.T,excellentVHSrlE$VHS.M,
method.reg = "PaBa",na.rm=T)
PB.rege@para
            #EST SE         LCI       UCI
  #Intercept 0.1 NA -0.6857143 0.100000
  #Slope     1.0 NA  1.0000000 1.071429
report_pb_para(PB.rege@para)

tiff("Figure1A_VHSmanual.tiff", width = 12, height = 12,units = "cm",
res=300,pointsize = 10)
MCResult.plot(PB.rege, equal.axis = TRUE, x.lab = "VHS-T", y.lab =
"VHS-M",
              ci.area = TRUE,
              main = "", sub = "", add.grid = FALSE, points.cex = 1,
              add.legend = T, add.cor = T)
dev.off()

#BlandAltman Analysis
attach(excellentVHSrlE)
blandr.statistics(VHS.T,VHS.M, sig.level=0.95)
  #t = -10.376, df = 160, p-value = < 2.2e-16
  #Bias: -0.09627329  ( -0.1145977  to  -0.07794887 ), SE 0.009 (SE
LoA 0.016)
  #ULoA:   0.1344831  ( 0.1031021  to  0.1658641 )
```

```
  #LLoA:   -0.3270297  ( -0.3584107  to  -0.2956487 )

#Save file with Fig1B
tiff("Figure1B_VHSmanual.tiff", width = 12, height = 12,units = "cm",
res=600,pointsize = 1)
grid::grid.draw(blandr.draw(VHS.T,VHS.M,method1name = "VHS-
T",method2name = "VHS-M",
                            plotTitle = "")+
                  theme_classic() +
                  labs(x = "Mean VHS", y= "Difference VHS.T-VHS.M"))
dev.off()



#####Repeat for VHS-M vs AI - Fig 3####
##Fig 3A Passing Bablok analysis for comparison of methods VHS
Modified vs AI
cor.test(excellentVHSrlE$VHS.M,excellentVHSrlE$VHS.Metron)##corr =
0.947, p-value < 2.2e-16, n= 1055
  # r= 0.948 CI 0.929-0.961, t = 37.413, df = 159, p-value < 2.2e-16

PB.rege_hai<- mcreg(excellentVHSrlE$VHS.M,excellentVHSrlE$VHS.Metron,
method.reg = "PaBa",na.rm=T)
PB.rege_hai@para
            # EST SE        LCI       UCI
  #Intercept -0.1 NA -0.1000000 0.5531066
  #Slope      1.0 NA  0.9411765 1.0000000


tiff("Figure3A_VHSai.tiff", width = 12, height = 12,units = "cm",
res=300,pointsize = 10)
MCResult.plot(PB.rege_hai, equal.axis = TRUE, x.lab = "VHS-M", y.lab =
"VHS-AI",
              ci.area = TRUE,
              main = "", sub = "", add.grid = FALSE, points.cex = 1,
              add.legend = T, add.cor = T)
dev.off()

##Bland Altman
blandr.statistics(VHS.M,VHS.Metron, sig.level=0.95)
  #t = t = 4.6102, df = 160, p-value = 8.183e-06
  #Bias: 0.09937888  ( 0.05680743  to  0.1419503 ) , SE 0.022 (SE LoA
0.037)
  #ULoA:    0.6354742  ( 0.5625696  to  0.7083788 )
  #LLoA:   -0.4367165  ( -0.509621  to  -0.3638119 )


#Save file with Fig3B
tiff("Figure3B_VHSmetron.tiff", width = 12, height = 12,units = "cm",
res=600,pointsize = 1)
```

```
grid::grid.draw(blandr.draw(VHS.M,VHS.Metron,method1name = "VHS-
M",method2name = "VHS-AI",
                            plotTitle = "")+
                  theme_classic() +
                  labs(x = "Mean VHS", y= "Difference VHS.M-VHS.AI"))
dev.off()


####Compare VHS-O to VHS-M - Fig 5#####
##Fig 5A Passing Bablok analysis for comparison of methods VHS
Traditional vs Modified
#Remove any rows with NA


cor.test(excellentVHSrlE$VHS.M,excellentVHSrlE$VHS.O) ##r=0.947, p<
2.2e-16, n=1054
 # r=0.939 CI 0.917-0.955, t = 34.299, df = 159, p-value < 2.2e-16
report_cor(excellentVHSrlE, VHS.M,VHS.O)

PB.regeo<- mcreg(excellentVHSrlE$VHS.M,excellentVHSrlE$VHS.O,
method.reg = "PaBa",na.rm=T)
PB.regeo@para
#              EST SE        LCI       UCI
#    Intercept   0 NA -0.1000000 0.9481621
#    Slope       1 NA  0.9128458 1.0000000
report_pb_para(PB.regeo@para)

tiff("Figure5A_VHSoriginal.tiff", width = 12, height = 12,units =
"cm", res=300,pointsize = 10)
MCResult.plot(PB.regeo, equal.axis = TRUE, x.lab = "VHS-M", y.lab =
"VHS-O",
              ci.area = TRUE,
              main = "", sub = "", add.grid = FALSE, points.cex = 1,
              add.legend = T, add.cor = T)
dev.off()

#BlandAltman Analysis Fig 5B
attach(excellentVHSrlE)
blandr.statistics(excellentVHSrlE$VHS.M,excellentVHSrlE$VHS.O,
sig.level=0.95)
  #t = 2.3697, df = 160, p-value = 0.01899
  #Bias: 0.0552795  ( 0.009209765  to  0.1013492 ) , SE 0.023 (SE LoA
0.040)
  #ULoA:    0.6354282  ( 0.5565327  to  0.7143236 )
  #LLoA:   -0.5248692  ( -0.6037646  to  -0.4459737 )
report_bland_altman(VHS.M,VHS.O)

tiff("Figure5B_VHSoriginal.tiff", width = 12, height = 12,units =
"cm", res=600,pointsize = 1)
grid::grid.draw(blandr.draw(excellentVHSrlE$VHS.M,excellentVHSrlE$VHS.
```

```
O,method1name = "VHS-M",method2name = "VHS-O",
                            plotTitle = "")+
                  theme_classic() +
                  labs(x = "Mean VHS", y= "Difference VHS.M-VHS.O"))
dev.off()

##Repeat for not poor
report_cor(notPoorVHSrlE,VHS.M,VHS.O)
attach(notPoorVHSrlE)
report_bland_altman(VHS.M,VHS.O)
PB.regedo<- mcreg(notPoorVHSrlE$VHS.M,notPoorVHSrlE$VHS.O, method.reg
= "PaBa",na.rm=T)
report_pb_para(PB.regedo@para)


###repeat for VLAS T vs M - Fig 2####
report_cor(excellentVLASrlE,VLAS.T,VLAS.M)
attach(excellentVLASrlE)
report_bland_altman(VLAS.T,VLAS.M)
report_pb_para(PB.rege_l@para)
##Fig 2A Passing Bablok analysis for comparison of methods VLAS
Traditional vs Modified
#Remove any rows with NA
cor.test(excellentVLASrlE$VLAS.T,excellentVLASrlE$VLAS.M) # Corr =
0.9735214 CI 0.9701740 0.9764976 , p<2.2e-16
  #r=0.982 CI 0.977-0.986, t = 78.844, df = 224, p-value < 2.2e-16

PB.rege_l<- mcreg(excellentVLASrlE$VLAS.T,excellentVLASrlE$VLAS.M,
method.reg = "PaBa",na.rm=T)
PB.rege_l@para
  #          EST SE   LCI UCI
  #Intercept   0 NA -0.05   0
  #Slope       1 NA  1.00   1

tiff("Figure2A_VLAS-manual.tiff", width = 12, height = 12,units =
"cm", res=300,pointsize = 10)
MCResult.plot(PB.rege_l, equal.axis = TRUE, x.lab = "VLAS-T", y.lab =
"VLAS-M",
              ci.area = TRUE,
              main = "", sub = "", add.grid = FALSE, points.cex = 1,
              add.legend = T, add.cor = T)
dev.off()

#BlandAltman Analysis
attach(excellentVLASrlE)
blandr.statistics(VLAS.T,VLAS.M, sig.level=0.95)
  #t = 7.4216, df = 225, p-value = 2.356e-12
  #Bias:  0.03672566  ( 0.02697441  to  0.04647692 )  , SE 0.005 (SE
LoA 0.008)
  #ULoA:    0.1825333  ( 0.165844  to  0.1992227 )
```

```
  #LLoA:    -0.109082  ( -0.1257714  to  -0.09239263 )

#Save file with Fig2B
tiff("Figure2B_VLAS-manual.tiff", width = 12, height = 12,units =
"cm", res=600,pointsize = 1)
grid::grid.draw(blandr.draw(VLAS.T,VLAS.M,method1name = "VLAS-
T",method2name = "VLAS-M",
                            plotTitle = "")+
                  theme_classic() +
                  labs(x = "Mean VLAS", y= "Difference VLAS.T-
VLAS.M"))
dev.off()


####Repeat for VLAS-M vs VLAS-AI - Fig 4####
report_cor(excellentVLASrlE,VLAS.M, VLAS.Metron)
attach(excellentVLASrlE)
report_bland_altman(VLAS.M, VLAS.Metron)
report_pb_para(PB.rege_lai@para)

##Fig 4A Passing Bablok analysis for comparison of methods VHS
Modified vs AI
cor.test(excellentVLASrlE$VLAS.M,excellentVLASrlE$VLAS.Metron)
  # r=0.844 CI 0.801-0.877, t = 23.504, df = 224, p-value < 2.2e-16
PB.rege_lai<-
mcreg(excellentVLASrlE$VLAS.M,excellentVLASrlE$VLAS.Metron, method.reg
= "PaBa",na.rm=T)
PB.rege_lai@para
  #           EST SE  LCI  UCI
#  Intercept 0.200 NA -0.1 0.36
#  Slope     0.875 NA  0.8 1.00



tiff("Figure4A_VLASai.tiff", width = 12, height = 12,units = "cm",
res=300,pointsize = 10)
MCResult.plot(PB.rege_lai, equal.axis = TRUE, x.lab = "VLAS-M", y.lab
= "VLAS-AI",
              ci.area = TRUE,
              main = "", sub = "", add.grid = FALSE, points.cex = 1,
              add.legend = T, add.cor = T)
dev.off()

##Bland Altman
blandr.statistics(VLAS.M,VLAS.Metron, sig.level=0.95)
  #t = 4.7715, df = 225, p-value = 3.289e-06
  #Bias:  0.06814159  ( 0.04000009  to  0.0962831 )  , SE 0.014 (SE
LoA 0.024)
  #ULoA:    0.4889333  ( 0.4407688  to  0.5370977 )
  #LLoA:    -0.3526501  ( -0.4008146  to  -0.3044856 )
```

```
#Save file with Fig4B
tiff("Figure4B_VLASmetron.tiff", width = 12, height = 12,units = "cm",
res=600,pointsize = 1)
grid::grid.draw(blandr.draw(VLAS.M,VLAS.Metron,method1name = "VLAS-
M",method2name = "VLAS-AI",
                            plotTitle = "")+
                  theme_classic() +
                  labs(x = "Mean VLAS", y= "Difference VLAS.M-
VLAS.AI"))
dev.off()



####Compare VLAS-O to VLAS-M - Fig 6#####

##Fig 6A Passing Bablok analysis for comparison of methods VHS
Traditional vs Modified
#Remove any rows with NA
cor.test(excellentVLASrlE$VLAS.M,excellentVLASrlE$VLAS.O) ##r=0.844,
p< 2.2e-16, n=820
  # r=0.794, 0.729-0.845, t = 16.49, df = 159, p-value < 2.2e-16
PB.regelo<- mcreg(excellentVLASrlE$VLAS.M,excellentVLASrlE$VLAS.O,
method.reg = "PaBa",na.rm=T)
PB.regelo@para
#          EST SE   LCI  UCI
#Intercept 0.1 NA 0.000 0.35
#Slope     1.0 NA 0.875 1.00

tiff("Figure6A_VLASoriginal.tiff", width = 12, height = 12,units =
"cm", res=300,pointsize = 10)
MCResult.plot(PB.regelo, equal.axis = TRUE, x.lab = "VLAS-M", y.lab =
"VLAS-O",
              ci.area = TRUE,
              main = "", sub = "", add.grid = FALSE, points.cex = 1,
              add.legend = T, add.cor = T)
dev.off()

#BlandAltman Analysis Fig 6B
blandr.statistics(excellentVLASrlE$VLAS.M,excellentVLASrlE$VLAS.O,
sig.level=0.95)
  #t = -3.297, df = 160, p-value = 0.001204
  #Bias:  -0.06459627  ( -0.1032892  to  -0.02590339 )  , SE 0.020 (SE
LoA 0.034)
  #ULoA:    0.4226569  ( 0.3563945  to  0.4889193 )
  #LLoA:    -0.5518494  ( -0.6181119  to  -0.485587 )



tiff("Figure6B_VLASoriginal.tiff", width = 12, height = 12,units =
```

```
"cm", res=600,pointsize = 1)
grid::grid.draw(blandr.draw(excellentVLASrlE$VLAS.M,excellentVLASrlE$V
LAS.O,method1name = "VLAS-M",method2name = "VLAS-O",
                            plotTitle = "")+
                  theme_classic() +
                  labs(x = "Mean VLAS", y= "Difference VLAS.M-
VLAS.O"))
dev.off()

####Now exclude poor rads, so include diagnostic and excellent####
setwd(notPoorDir)
#VHS n=161
notPoorVHSrlE<- full%>%
  filter(OverallRLVHSlandmarks %in% c("E","D"))
#VLAS n=161
notPoorVLASrlE<- full%>%
  filter(OverallRLVLASlandmarks %in% c("E","D"))
#####VHS-traditional vs modified - Fig 1 #####
##Fig 1A Passing Bablok analysis for comparison of methods VHS
Traditional vs Modified
report_cor(notPoorVHSrlE,VHS.T, VHS.M)
attach(notPoorVHSrlE)
report_bland_altman(VHS.T, VHS.M)
report_pb_para(PB.regP@para)
#Remove any rows with NA
cor.test(notPoorVHSrlE$VHS.T,notPoorVHSrlE$VHS.M)
  #r= 0.994 CI 0.993-0.994, t = 246.56, df = 769, p-value < 2.2e-16
PB.regP<- mcreg(notPoorVHSrlE$VHS.T,notPoorVHSrlE$VHS.M, method.reg =
"PaBa",na.rm=T)
PB.regP@para
  #           EST       SE        LCI      UCI
  #Intercept -0.5411765 NA -0.6402414 -0.400000
  #Slope      1.0588235 NA  1.0454545  1.066782


tiff("Figure1A_VHSmanual.tiff", width = 12, height = 12,units = "cm",
res=300,pointsize = 10)
MCResult.plot(PB.regP, equal.axis = TRUE, x.lab = "VHS-T", y.lab =
"VHS-M",
              ci.area = TRUE,
              main = "", sub = "", add.grid = FALSE, points.cex = 1,
              add.legend = T, add.cor = T)
dev.off()

#BlandAltman Analysis
attach(notPoorVHSrlE)
blandr.statistics(VHS.T,VHS.M, sig.level=0.95)
  #t = -20.763, df = 770, p-value = < 2.2e-16
  #Bias: -0.0997406  ( -0.1091707  to  -0.09031046 ), SE 0.005 (SE LoA
0.008)
```

```
  #ULoA:   0.1616983  ( 0.1455752  to  0.1778214 )
  #LLoA:    -0.3611795  ( -0.3773026  to  -0.3450563 )

#Save file with Fig1B
tiff("Figure1B_VHSmanual.tiff", width = 12, height = 12,units = "cm",
res=600,pointsize = 1)
grid::grid.draw(blandr.draw(VHS.T,VHS.M,method1name = "VHS-
T",method2name = "VHS-M",
                            plotTitle = "")+
                  theme_classic() +
                  labs(x = "Mean VHS", y= "Difference VHS.T-VHS.M"))
dev.off()



#####Repeat for VHS-M vs AI - Fig 3####
report_cor(notPoorVHSrlE,VHS.M, VHS.Metron)
attach(notPoorVHSrlE)
report_bland_altman(VHS.M, VHS.Metron)
report_pb_para(PB.regP_hai@para)
##Fig 3A Passing Bablok analysis for comparison of methods VHS
Modified vs AI
cor.test(notPoorVHSrlE$VHS.M,notPoorVHSrlE$VHS.Metron)##corr = 0.947,
p-value < 2.2e-16, n= 1055
# r= 0.951 CI 0.943-0.957, t = 84.996, df = 769, p-value < 2.2e-16

PB.regP_hai<- mcreg(notPoorVHSrlE$VHS.M,notPoorVHSrlE$VHS.Metron,
method.reg = "PaBa",na.rm=T)
PB.regP_hai@para
  #              EST      SE         LCI       UCI
  #Intercept 0.4136364 NA -0.1000000 0.6928571
  #Slope     0.9545455 NA  0.9285714 1.0000000


tiff("Figure3A_VHSai.tiff", width = 12, height = 12,units = "cm",
res=300,pointsize = 10)
MCResult.plot(PB.regP_hai, equal.axis = TRUE, x.lab = "VHS-M", y.lab =
"VHS-AI",
              ci.area = TRUE,
              main = "", sub = "", add.grid = FALSE, points.cex = 1,
              add.legend = T, add.cor = T)
dev.off()

##Bland Altman
blandr.statistics(VHS.M,VHS.Metron, sig.level=0.95)
  #t = 7.4493, df = 770, p-value = 2.522e-13
  #Bias: 0.08754864  ( 0.06447761  to  0.1106197 ), SE 0.012 (SE LoA
0.020)
  #ULoA:    0.727164  ( 0.6877184  to  0.7666095 )
  #LLoA:   -0.5520667  ( -0.5915122  to  -0.5126212 )
```

```
#Save file with Fig3B
tiff("Figure3B_VHSmetron.tiff", width = 12, height = 12,units = "cm",
res=600,pointsize = 1)
grid::grid.draw(blandr.draw(VHS.M,VHS.Metron,method1name = "VHS-
M",method2name = "VHS-AI",
                            plotTitle = "")+
                  theme_classic() +
                  labs(x = "Mean VHS", y= "Difference VHS.M-VHS.AI"))
dev.off()


###repeat for VLAS T vs M - Fig 2####
report_cor(notPoorVLASrlE,VLAS.T, VLAS.M)
attach(notPoorVLASrlE)
report_bland_altman(VLAS.T, VLAS.M)
report_pb_para(PB.regP_l@para)
##Fig 2A Passing Bablok analysis for comparison of methods VLAS
Traditional vs Modified
#Remove any rows with NA
cor.test(notPoorVLASrlE$VLAS.T,notPoorVLASrlE$VLAS.M) # Corr =
0.9735214 CI 0.9701740 0.9764976 , p<2.2e-16
  #r=0.973 CI 0.969-0.976, t = 123.41, df = 863, p-value < 2.2e-16
PB.regP_l<- mcreg(notPoorVLASrlE$VLAS.T,notPoorVLASrlE$VLAS.M,
method.reg = "PaBa",na.rm=T)
PB.regP_l@para
  #          EST SE   LCI UCI
  #Intercept  0 NA   0   0
  #Slope       1 NA   1   1

tiff("Figure2A_VLAS-manual.tiff", width = 12, height = 12,units =
"cm", res=300,pointsize = 10)
MCResult.plot(PB.regP_l, equal.axis = TRUE, x.lab = "VLAS-T", y.lab =
"VLAS-M",
              ci.area = TRUE,
              main = "", sub = "", add.grid = FALSE, points.cex = 1,
              add.legend = T, add.cor = T)
dev.off()

#BlandAltman Analysis Fig 2B
attach(notPoorVLASrlE)
blandr.statistics(VLAS.T,VLAS.M, sig.level=0.95)
  #t = 11.35, df = 864, p-value = < 2.2e-16
  #Bias:  0.04219653  ( 0.03489963  to  0.04949343 )   , SE 0.004 (SE
LoA 0.006)
  #ULoA:    0.2565082  ( 0.2440329  to  0.2689834 )
  #LLoA:    -0.1721151  ( -0.1845903  to  -0.1596398 )

#Save file with Fig2B
```

```
tiff("Figure2B_VLAS-manual.tiff", width = 12, height = 12,units =
"cm", res=600,pointsize = 1)
grid::grid.draw(blandr.draw(VLAS.T,VLAS.M,method1name = "VLAS-
T",method2name = "VLAS-M",
                            plotTitle = "")+
                  theme_classic() +
                  labs(x = "Mean VLAS", y= "Difference VLAS.T-
VLAS.M"))
dev.off()


####Repeat for VLAS-M vs VLAS-AI - Fig 4####
report_cor(notPoorVLASrlE,VLAS.M, VLAS.Metron)
attach(notPoorVLASrlE)
report_bland_altman(VLAS.M, VLAS.Metron)
report_pb_para(PB.regP_lai@para)
##Fig 4A Passing Bablok analysis for comparison of methods VHS
Modified vs AI
cor.test(notPoorVLASrlE$VLAS.M,notPoorVLASrlE$VLAS.Metron)##corr =
0.811, p-value < 2.2e-16, n= 1055
  # r=0.8454 CI 0.834-0.871, t = 48.124, df = 863, p-value < 2.2e-16
PB.regP_lai<- mcreg(notPoorVLASrlE$VLAS.M,notPoorVLASrlE$VLAS.Metron,
method.reg = "PaBa",na.rm=T)
PB.regP_lai@para
  #            EST       SE LCI  UCI
  #  Intercept 0.3000000 NA 0.2 0.360
  #  Slope     0.8333333 NA 0.8 0.875



tiff("Figure4A_VLASai.tiff", width = 12, height = 12,units = "cm",
res=300,pointsize = 10)
MCResult.plot(PB.regP_lai, equal.axis = TRUE, x.lab = "VLAS-M", y.lab
= "VLAS-AI",
              ci.area = TRUE,
              main = "", sub = "", add.grid = FALSE, points.cex = 1,
              add.legend = T, add.cor = T)
dev.off()

##Bland Altman
blandr.statistics(VLAS.M,VLAS.Metron, sig.level=0.95)
  #t = 7.7483, df = 864, p-value = 2.62e-14
  #Bias:  0.06473988  ( 0.04834073  to  0.08113904 )  , SE 0.008 (SE
LoA 0.014)
  #ULoA:    0.5463866  ( 0.5183496  to  0.5744237 )
  #LLoA:    -0.4169069  ( -0.4449439  to  -0.3888698 )

#Save file with Fig4B
tiff("Figure4B_VLASmetron.tiff", width = 12, height = 12,units = "cm",
res=600,pointsize = 1)
```

```
grid::grid.draw(blandr.draw(VLAS.M,VLAS.Metron,method1name = "VLAS-M",method2name = "VLAS-AI",
                            plotTitle = "")+
                  theme_classic() +
                  labs(x = "Mean VLAS", y= "Difference VLAS.M-VLAS.AI"))
dev.off()


###Report Comparisons of Left vs Right ###
#Once we have the AI measurements on the Left lateral rads
#VHS-AI
report_cor(full,VHS.Metron, LL.VHS.Metron)
attach(full)
report_bland_altman(VHS.Metron, LL.VHS.Metron)
PB.regP_LR_VHS<- mcreg(full$VHS.Metron,full$LL.VHS.Metron, method.reg = "PaBa",na.rm=T)
report_pb_para(PB.regP_LR_VHS@para)

#VLAS-AI - repeat above
report_cor(full,VLAS.Metron, LL.VLAS.Metron)
attach(full)
report_bland_altman(VLAS.Metron, LL.VLAS.Metron)
PB.regP_LR_VLAS<- mcreg(full$VLAS.Metron,full$LL.VLAS.Metron, method.reg = "PaBa",na.rm=T)
report_pb_para(PB.regP_LR_VLAS@para)


###Report Comparisons of Left Modified vs AI ###
#Once we have the AI measurements on the Left lateral rads
#VHS-AI
report_cor(full,LL.VHS.human.modified, LL.VHS.Metron)
attach(full)
report_bland_altman(LL.VHS.human.modified, LL.VHS.Metron)
PB.regP_Left_VHS<-
mcreg(full$LL.VHS.human.modified,full$LL.VHS.Metron, method.reg = "PaBa",na.rm=T)
report_pb_para(PB.regP_Left_VHS@para)

#VLAS-AI - repeat above
report_cor(full,LL.VLAS.human.modified, LL.VLAS.Metron)
attach(full)
report_bland_altman(LL.VLAS.human.modified, LL.VLAS.Metron)
PB.regP_Left_VLAS<-
mcreg(full$LL.VLAS.human.modified,full$LL.VLAS.Metron, method.reg = "PaBa",na.rm=T)
report_pb_para(PB.regP_Left_VLAS@para)


####Next steps####
```

```r
#Do histogram of differences of human and AI with 95% CI
#Make function for this:
plot_diff_percentiles <- function(x, y,
                                  title = "Histogram of Differences",
                                  xlab = "Difference (x - y)",
                                  col_fill = "lightgray",
                                  border_col = "black",
                                  breaks = "Sturges") {
  # Remove NA pairs
  df <- na.omit(data.frame(x, y))
  diff <- df$x - df$y

  # Create histogram object (no plot yet)
  h <- hist(diff, breaks = breaks, plot = FALSE)

  # Define axis limits with a small buffer
  xlim <- range(h$breaks)
  ylim <- c(0, max(h$counts) * 1.05)

  # Plot manually to control axes
  plot(h,
       main = title,
       xlab = xlab,
       ylab = "Frequency",
       col = col_fill,
       border = border_col,
       xlim = xlim,
       ylim = ylim,
       axes = FALSE,
       yaxs = "i",   # ensure y-axis starts at 0 (touches bars)
       xaxs = "i")   # ensure x-axis touches bars)

  # Draw axes in full contact with histogram
  axis(1, lwd = 2, pos =0)  # X-axis
  axis(2, lwd = 2)  # Y-axis
 # box(lwd = 2)      # Frame box

  # Compute percentiles
  p2.5  <- quantile(diff, 0.025)
  p5    <- quantile(diff, 0.05)
  p95   <- quantile(diff, 0.95)
  p97.5 <- quantile(diff, 0.975)

  # Add percentile lines
  abline(v = c(p5, p95), col = "red", lty = 2, lwd = 2)
  abline(v = c(p2.5, p97.5), col = "blue", lty = 2, lwd = 2)


}
```

```
#------------------------------------------
# Use: VHS-M and VHS-AI with 95%CI
#------------------------------------------

#Histogram of the difference between vertebral heart size (VHS) of
cardiologists and algorithm. The red lines indicate the 5th percentile
and 95th percentile. The blue dashed lines represent the 2.5th
percentile and the 97.5th percentile
plot_diff_percentiles(full$VHS.M, full$VHS.Metron,
                      title = "",
                      xlab = "Difference (VHS.M - VHS.AI)",
                      breaks=40)

#Histogram of the difference between vertebral left atrial size (VLAS)
of cardiologists and algorithm. The red lines indicate the 5th
percentile and 95th percentile. The blue dashed lines represent the
2.5th percentile and the 97.5th percentile
plot_diff_percentiles(full$VLAS.M, full$VLAS.Metron,
                      title = "",
                      xlab = "Difference (VLAS.M - VLAS.AI)",
                      breaks=40)


abs_diff_percentile <-function(x, y,
                                    percentile = 0.95,
                                    n_boot = 10000,
                                    na.rm = TRUE,
                                    conf = 0.95,
                                    seed = 123) {
  # Check length
  if(length(x) != length(y)) stop("x and y must be the same length")

  # Remove NA pairs if requested
  if(na.rm) {
    df <- na.omit(data.frame(x, y))
    x <- df$x
    y <- df$y
  }

  # Absolute differences
  abs_diff <- abs(x - y)

  # Function to compute percentile
  perc_fun <- function(data, indices) {
    quantile(data[indices], percentile)
  }

  # Load boot package
  if(!requireNamespace("boot", quietly = TRUE)) {
```

```
    stop("Package 'boot' is required. Please install it first.")
  }
  library(boot)

  # Bootstrapping
  set.seed(seed)
  boot_res <- boot::boot(data = abs_diff, statistic = perc_fun, R =
n_boot)

  # Confidence interval
  ci <- boot::boot.ci(boot_res, type = "perc", conf = conf)

  # Return list with percentile and CI
  list(
    percentile = quantile(abs_diff, percentile),
    CI_lower = ci$percent[4],
    CI_upper = ci$percent[5]
  )
}

#-------------------------------------------
# USE
#-------------------------------------------
abs_diff_percentile(full$VHS.M, full$VHS.Metron)
abs_diff_percentile(full$VLAS.M, full$VLAS.Metron)


#Attempt to make a grid of plots
```
